# Influence of APOE Genotype on Alzheimer's Disease CSF Biomarkers in a Spanish Population

**DOI:** 10.1155/2016/1390620

**Published:** 2016-03-22

**Authors:** J. A. Monge-Argilés, R. Gasparini-Berenguer, M. Gutierrez-Agulló, C. Muñoz-Ruiz, J. Sánchez-Payá, C. Leiva-Santana

**Affiliations:** ^1^Department of Neurology, University General Hospital of Alicante, Avenida Pintor Baeza 12, 03010 Alicante, Spain; ^2^Genetics Laboratory, University General Hospital of Alicante, Avenida Pintor Baeza 12, 03010 Alicante, Spain; ^3^Immunology Laboratory, University General Hospital of Alicante, Avenida Pintor Baeza 12, 03010 Alicante, Spain; ^4^Department of Preventive Medicine, University General Hospital of Alicante, Avenida Pintor Baeza 12, 03010 Alicante, Spain

## Abstract

*Objectives*. To evaluate the association between apolipoprotein E (APOE) genotype and cerebrospinal fluid (CSF) levels of Alzheimer's disease (AD) biomarkers and to study the influence of APOE genotype on the development of AD in a Spanish population.* Material and Methods*. The study comprised 29 amnestic mild cognitive impairment (MCI) patients and 27 control subjects. Using ELISA methodology, CSF biomarkers and tau/A*β* ratios were obtained. ANOVA and adjusted odds ratios were calculated.* Results*. We observed the effect of APOE genotype and age on CSF AD variables. The progression to AD was more clearly influenced by CSF AD variables than by age or APOE status.* Conclusions*. APOE status influences CSF AD variables. However, the presence of APOE *ε*4 does not appear to be a deterministic factor for the development of AD, because CSF variables have a greater influence on progression to the disease. These results confirm previous observations and, to our knowledge, are the first published in a Spanish population.

## 1. Introduction

Alzheimer's disease (AD) is the most common form of dementia in the elderly. Genetic, pathological, and functional studies have shown that an imbalance between production and clearance of amyloid-*β* (A*β*) peptides in the brain results in accumulation and aggregation of A*β*. Aggregates of toxic A*β* in the form of soluble oligomers, intraneuronal A*β*, and amyloid plaques injure synapses and ultimately cause degeneration and dementia [1].

The APOE gene regulates lipid homeostasis by mediating lipid transport from one tissue or cell to another. The human APOE gene exists as three polymorphic alleles (*ε*2, *ε*3, and *ε*4) and it is known to play an important role in A*β* metabolism [1].

In the National Institute of Aging-Alzheimer's Association (NIA-AA) criteria for the diagnosis of AD, CSF biomarkers have been accepted as evidence of the pathophysiological process, mostly for research purposes [2]. However, there is no consensus on what constitutes a “CSF Alzheimer profile” [3], probably because different factors influence the absolute values of these biomarkers. Although these factors include age [4], there is some controversy about the influence of APOE genotype on CSF biomarkers. Some authors find no association between them [5, 6] while others find a clear relationship [1, 7, 8].

When it has been proved, the relation was found in American and European populations, but the incidence and prevalence rates of AD varied between countries [9]. For example, incidence ranges from 0.04 per 1000 person-years in the UK (people aged 45–65 years) to 16.8 per 1000 person-years in the USA (people aged 65 years and over). Prevalence ranges from Spain (6.7% in those older than 75 years old) to USA (4.9% in those older than 70 years old) [9]. The reasons for this are not completely understood, but the APOE genotype should be a factor to exclude. We hypothesized that the APOE genotype influences CSF AD biomarker levels in a Spanish population, as it is does in other populations. To our knowledge, this is the first publication on this matter.

## 2. Material and Methods

### 2.1. Study Design and Subjects

This longitudinal study included 29 consecutive amnestic MCI patients, according to the Petersen criteria 2006 [10], who attended the cognitive deterioration out-patient clinic of the General Hospital of Alicante in 2011. All participants underwent physical and neurological examination, neuropsychological studies, cerebral magnetic resonance imaging (MRI), blood tests, APOE genotyping, and lumbar puncture (LP) for assessment of CSF AD biomarkers. These patients were reviewed every six months for two years regarding the development of dementia, using both the NIA-AA criteria [2] and the Global Deterioration Scale (GDS) by Reisberg.

A control group comprising 27 consecutive subjects without subjective memory loss or known cognitive deterioration was included. These subjects were patients due to undergo spinal anaesthesia for orthopaedic or nonmalignant urological conditions. Clinical details, including blood test results, were collected. A neuropsychological study and APOE genotyping were performed a few days after the relevant surgical procedure. These subjects were then invited to attend the cognitive impairment out-patient clinic annually for follow-up over two years. Both groups were matched for age, gender, and education.

### 2.2. Inclusion Criteria

The inclusion criteria required that the patients be over the age of 55 with concordant clinical and neuropsychological diagnosis. In the control group, no patients had subjective memory loss, all mini mental state examination (MMSE) results were above 27, and scores on the informant questionnaire on cognitive decline in the elderly (IQCODE) were below 78. The neuropsychological criteria for the MCI group were MMSE scores between 23 and 26 and IQCODE above 78. Informed consent was obtained before inclusion and also before LP.

### 2.3. Exclusion Criteria

Exclusion criteria were the presence of dementia or other neurological, psychiatric, or medical disorders which could provoke cognitive deterioration, anticoagulant therapy, failure to obtain informed consent, or a score greater than five using the Depression Scale by Yesavage.

### 2.4. Procedures

The neurologist responsible for each MCI patient diagnosed single or multiple domain amnestic MCI according to Petersen's criteria [10]. Following this, a neuropsychological report enabled reclassification of the MCI patients. The neuropsychological examination included the following: MMSE, IQCODE, Rey Auditory Verbal Learning, Trail Making Test, and the Geriatric Depression Scale by Yesavage. With these tests we evaluated memory, language, executive function, attention, and visuoconstructive capacity. Alteration of a function was defined as a *Z* result of −1.5 or less, which was at least 1.5 standard deviations below the mean of the control subjects, in at least one of the tests used to evaluate that function. The same neuropsychological tests were performed in the control group and in the patient group. The GDS and the NIA-AA clinical criteria were used for the diagnosis of AD at the two-year follow-up.

### 2.5. Extraction and Analysis of CSF

The extraction of CSF was performed between January and December 2011. The samples were collected between 10:00 a.m. and 2:00 p.m. In patients with MCI, the LP was performed by their own neurologist with a 20 × 3.5 gauge needle. The CSF sample was collected in standard tubes and centrifuged, if minimally sanguinolent, before being frozen. Obvious sanguinolent CSF was discarded. The CSF (±1 mL) of control subjects was obtained in the operating theatre by the anaesthetist performing the spinal anaesthesia. After LP, all patients were advised to avoid Valsalva manoeuvres for at least three days.

### 2.6. Quantification of CSF Biomarker Levels

Quantification was performed using ELISA methodology and INNOTEST reagents from Innogenetics (Ghent, Belgium). The details of this reagent combination for immunoassay and analytic platform have been published previously [11]. All samples were analysed simultaneously after recruitment was completed and blindly regarding the clinical details.

### 2.7. Analysis of APOE Genotype

The APOE allele status was determined by genotyping with polymerase chain reaction and restriction fragment length polymorphism by gel electrophoresis, as described previously [12]. All serum samples were kept frozen at −70°C until assay.

### 2.8. Study Variables

Variables were levels of A*β*_1–42_, T-tau, and p-tau_181p_ proteins in the CSF as well as the T-tau/A*β*_1–42_ and p-tau/A*β*_1–42_ ratios. These latter variables are frequently used by many authors and appear to reflect the relationship between the two pathophysiological mechanisms of the disease (amyloid and tau). We classified each subject by APOE genotype as either *ε*2 or *ε*4 (homozygous or heterozygous) and *ε*3 (only homozygous).

### 2.9. Statistical Analysis

To study the association between APOE genotype and different biomarkers adjusted for age, the analysis of variance (ANOVA) was used for multiple variables. To quantify the association between APOE genotype and progression of Alzheimer's disease-adjusted levels of each biomarker and age, we used a multiple logistic regression model to calculate the adjusted odds ratios (OR) and their 95% confidence intervals. The level of statistical significance used in hypothesis testing was *p* < 0.05. Data analysis was performed with IBM-SPSS version 19.1 software (IBM Corp., Armonk, NY).

### 2.10. Ethical Criteria

The two pharmaceutical companies who contributed to this project had no role in the study design, data collection and interpretation, or drafting of the final report. This study was fully approved by the University General Hospital of Alicante Ethical Committee.

## 3. Results

The demographic, genetic, and clinical characteristics of the study population are shown in Table 1. Overall, 17.8% were genotype *ε*2, 55.3% were *ε*3, and 26.7% were *ε*4. At inclusion, there were significant differences in MMSE and IQCODE between both groups. No differences were found in age, medical history, or education level.

The APOE genotype had a clear influence on all CSF AD variables, after the exclusion of the influence of age (adjusted significance level), as indicated in Table 2. As expected, the *ε*4 subjects showed lower A*β*42 and higher protein levels of T-tau and p-tau and ratios of T-tau and p-tau to A*β*42 than the other genotypes.  Table 3 shows that age influenced almost all the CSF variables, but this influence was lost after adjustment.

Table 4 shows the differences in CSF AD variables after the clinical diagnosis at the two-year follow-up. As expected, patients who had developed AD at the follow-up had lower A*β*42 levels and higher protein levels of T-tau and p-tau and ratios of T-tau and p-tau to A*β*42 at inclusion.

The influence of the APOE genotype and age on progression to AD at the two-year follow-up is shown in Table 5. Older patients and those with genotype *ε*4 had higher adjusted OR.

Table 6 shows the influence of CSF AD variables on progression to AD at the two-year follow-up, after adjusting for APOE genotype and age. T-tau protein levels and ratios had the highest adjusted OR for progression to AD, whereas the A*β*42 levels had the lowest.

Cerebral MRI excluded structural lesions. There were no differences in white matter hyperintensities or degree of cerebral atrophy between groups.

## 4. Discussion

In our results, the APOE *ε*4 status was associated with lower CSF A*β*42, as well as higher CSF T-tau and p-tau protein levels and tau/A*β*42 ratios, in the early prodromal stages of AD patients and in the control subjects, taking into account the influence of age. In the last few years, the APOE *ε*4 status has been accepted as important in CSF AD biomarker levels [4], as witnessed in our findings as well. However, the APOE genotype showed a clearer influence on all the variables obtained from the CSF AD analysis, especially on A*β*42 levels and ratios, as published previously [7, 8].

The influence of the APOE *ε*4 genotype on CSF AD biomarkers has been widely described [13–16], but there is no consensus as some authors find no association between them [5, 6]. Our results agree with the first group of publications. It is currently accepted that APOE isoforms differentially regulate A*β* aggregation and clearance in the brain and have distinct functions in regulating brain lipid transport, glucose metabolism, neuronal signalling, neuroinflammation, and mitochondrial function [1, 7]. The effect of the APOE genotype on amyloid deposition has been shown in middle-aged and older cognitively healthy adults [8], as well as in patients with MCI and AD [17–22].

All these basic, clinical, and laboratory data underscore the importance of considering the APOE genotype when evaluating CSF biomarkers, because it could be at least partially responsible for the observed disease heterogeneity [21]. A gene described recently as SUCLG2 (Succinyl-CoA Ligase) appears to determine CSF A*β* levels and attenuate cognitive decline in AD [23]. Moreover, other genes have been found to enhance the risk of sporadic AD, such as phosphatidylinositol-binding clathrin assembly protein (PICALM) [24], or the translocase of the outer mitochondrial membrane (TOMM40) [25, 26], but, to our knowledge, their influence on CSF AD biomarkers has not yet been studied.

Based on our results, the APOE genotype has less influence than every CSF AD variables in the development of AD in MCI patients and control subjects, except A*β*42. These data agree with recent publications showing that the APOE genotype did not significantly improve the prediction of AD in MCI patients [22, 27]. However, there is broad evidence of the ability of CSF AD biomarkers to improve the prediction of AD in MCI patients [11, 28–32] and elderly control subjects [33, 34], despite the difficulties in achieving global standardization measures [35] and the optimal method to evaluate the results [3].

In this study, the progression rate of control subjects to AD was near the expected rate (3.5% per year). However, the progression rate of MCI patients to AD was slightly higher than what would be expected (about 40% at two years) and highlights the importance of the utilisation of that clinical entity in these studies.

The present study has some limitations. First, even though our sample size was small, we nevertheless obtained results similar to those of previous studies. Second, we did not evaluate all known AD biomarkers nor did we use PIB-PET or advanced MRI techniques. Finally, we did not have pathological confirmation of the diagnosis of AD.

In conclusion, the APOE *ε*4 status is associated with lower CSF A*β*42 as well as higher CSF T-tau and p-tau protein levels and tau/A*β* ratios, in patients in the early prodromal stages of AD and in control subjects. However, the presence of APOE *ε*4 does not seem to be a deterministic factor for the development of AD. To the best of our knowledge, these are the first results described in a Spanish population.

## Figures and Tables

**Table 1:** Demographic, clinical, and genetic characteristics of the study population.

	MCI patients	Control subjects	Significance level
Number of cases	29	27	n.s.
Gender, male (%)	38	44	n.s.
Age (mean ± SD)	70.34 ± 7.3	68.74 ± 7.5	n.s.
Medical history			
HTA	16	17	—
DM	2	8	
HPL	17	18	
Depression	7	6	
MMSE Folstein	24 ± 0.8	28.48 ± 2.5	0.05
IQCODE	82.89 ± 3.5	52.62 ± 14.9	0.01
Progression at 2 years			
Stable normal	—	22	—
Stable MCI	17	—	
Develop MCI	—	3	
Develop AD	12 (*ε*4 = 8, *ε*3 = 4)	2 (*ε*3 = 1, *ε*2 = 1)	
APOE genotype		
*ε*2	2	8
*ε*3	14	18
*ε*4	13	1
Education (years)	5	6	n.s.

SD = standard deviation. HTA = hypertension. DM = diabetes mellitus. HPL = hyperlipidemia. MMSE = mini mental status examination. IQCODE = informant questionnaire on cognitive decline in the elderly. MCI = mild cognitive impairment. AD = Alzheimer's disease.

**Table 2:** APOE genotype influence on CSF AD biomarker variables.

CSF variables	APOE genotype	Number of subjects	Mean ± SD	Significance level	Adjusted significance level
A*β* protein (pg/mL)	*ε*2	10	1320.50 ± 489.51		
	*ε*3	31	1178.23 ± 428.40	0.0001	0.001
	*ε*4	15	656.80 ± 201.96		

T-tau protein (pg/mL)	*ε*2	10	207.00 ± 110.89		
	*ε*3	31	259.39 ± 130.60	0.0001	0.004
	*ε*4	15	488.27 ± 284.34		

			Median (*P*25–*P*75)		

p-tau protein (pg/mL)	*ε*2	10	38 (30.27–42.73)		
	*ε*3	31	44 (42.97–63.94)	0.06^*∗*^	0.02
	*ε*4	15	71 (57.0–95.17)		

Ratio T-tau/A*β*	*ε*2	10	0.14 (0.08–0.26)		
	*ε*3	31	0.18 (0.19–0.34)	0.18^*∗*^	0.0001
	*ε*4	15	0.74 (0.55–1.06)		

Ratio p-tau/A*β*	*ε*2	10	0.03 (0.02–0.03)		
	*ε*3	31	0.03 (0.03–0.07)	0.09^*∗*^	0.0001
	*ε*4	15	0.11 (0.08–0.17)		

ANOVA 1 factor. ^*∗*^Chi-square. SD: standard deviation.

**Table 3:** Influence of age on CSF AD variables.

CSF variables	Age	Number of patients	Mean ± SD	Significance level	Adjusted significance level
A*β* protein (pg/mL)	≥75 <75	16 40	858.63 ± 457.41 1146.1 ± 444.34	0.03	0.06

T-tau protein (pg/mL)	≥75 <75	16 40	381.75 ± 172.46 283.18 ± 218.34	0.11	0.38

			Median (*P*25–*P*75)		

p-tau protein (pg/mL)	≥75 <75	16 40	59 (52.0–88.68) 40 (42.1–60.15)	0.01^*∗*^	0.21

Ratio T-tau/A*β*	≥75 <75	16 40	0.42 (0.35–0.74) 0.16 (0.21–0.45)	0.007^*∗*^	0.26

Ratio p-tau/A*β*	≥75 <75	16 40	0.07 (0.05–0.15) 0.03 (0.04–0.07)	0.004^*∗*^	0.09

Student's *t*-test. ^*∗*^Mann-Whitney *U* test.

**Table 4:** CSF AD variables after clinical diagnosis at two-year follow-up.

CSF variables	Diagnosis	Number of patients	Mean ± S.D.	Significance level
A*β* protein (pg/mL)	AD No AD	14 42	743.36 ± 292.64 1170.83 ± 462.0	0.002

T-tau protein (pg/mL)	AD No AD	14 42	511.21 ± 271.6 244.71 ± 131.75	0.0001

			Median (*P*25–*P*75)	

p-tau protein (pg/mL)	AD No AD	14 42	76.0 (63.53–106.75) 41.5 (40.3–53.94)	0.0001^*∗*^

Ratio T-tau/A*β*	AD No AD	14 42	0.83 (0.52–1.08) 0.16 (0.09–0.32)	0.0001^*∗*^

Ratio p-tau/A*β*	AD No AD	14 42	0.11 (0.08–0.18) 0.03 (0.03–0.05)	0.0001^*∗*^

Student's *t*-test. ^*∗*^Mann-Whitney *U* test.

**Table 5:** Influence of APOE genotype and age over the progression to AD.

	Frequency of progression to AD	Crude odds ratio (95% CI)	Adjusted odds ratio (95% CI)
Genotype			
*ε*2	10.0% (1/10)	1	1
*ε*3	12.9% (4/31)	1.3 (0.1–13.5)	0.9 (0.08–10.5)
*ε*4	60.0% (9/15)	13.5 (1.3–136.0)	4.7 (0.3–69.9)
Age			
≥75	43.8% (7/16)	3.7 (1.1–13.2)	2.9 (0.6–13.4)
<75	17.5% (7/40)	1	1

**Table 6:** Influence of CSF AD variables on progression to AD.

	Frequency of progression to AD	Crude odds ratio (95% CI)	Adjusted odds ratio (95% CI)
A*β* protein levels			
≤710 (pg/mL)	60.0% (9/15)	10.8 (2.7–43.)	3.8 (0.6–22.1)
≥711 (pg/mL)	12.2% (5/41)	1	1
T-tau protein levels			
≤421 (pg/mL)	71.4% (10/14)	1	1
≥422 (pg/mL)	9.5% (4/42)	23.7 (5.0–112.0)	13.9 (2.2–87.0)
p-tau protein levels			
≤63 (pg/mL)	11.6% (5/43)	1	1
≥64 (pg/mL)	69.2% (9/13)	17.1 (3.8–76.8)	10.34 (1.84–58.14)
T-tau/A*β* ratio			
<0.54	9.5% (4/42)	1	1
≥0.54	71.4% (10/14)	23.75 (5.0–112.0)	19.83 (2.64–148.56)
p-tau/A*β* ratio			
<0.10	9.5% (4/42)	1	1
≥0.10	71.4% (10/14)	23.75 (5.0–112.0)	13.91 (2.18–88.74)
